# The Benefit of Sirolimus Maintenance Immunosuppression and Rabbit Antithymocyte Globulin Induction in Liver Transplant Recipients That Develop Acute Kidney Injury in the Early Postoperative Period

**DOI:** 10.1155/2015/926168

**Published:** 2015-03-11

**Authors:** Benjamin T. Duhart, Winston A. Ally, Amy G. Krauss, Joanna Q. Hudson, James D. Eason, Vinaya Rao, Jason M. Vanatta

**Affiliations:** ^1^University of Tennessee College of Pharmacy, Memphis, TN 38163, USA; ^2^Methodist University Hospital Transplant Institute, University of Tennessee Health Science Center, Memphis, TN 38103, USA; ^3^University of Virginia Medical Center, Charlottesville, VA 22908, USA

## Abstract

Published data are limited describing renal outcomes in orthotopic liver transplant (OLT) recipients prescribed sirolimus (SRL) maintenance immunosuppression (MIS) and rabbit antithymocyte globulin (rATG) induction. We investigated whether SRL MIS and rATG induction facilitated recovery of acute kidney injury in the early postoperative period. This retrospective descriptive study screened 308 consecutive OLTs performed between 2006 and 2009. All patients received rATG induction with steroid avoidance. MIS consisted of SRL or TAC with mycophenolate mofetil. A total of 197 patients were included: 168 (85%) received TAC and 29 (15%) received SRL for a median of 365 days. Demographics were similar between groups except for a higher incidence of pretransplant renal dysfunction in the SRL recipients (SRL 59% versus TAC 21%; *P* < 0.05). The eGFR was significantly (*P* < 0.05) higher for all time points in the TAC group with the exception of month 2. However, improvement in eGFR was significantly (*P* < 0.05) greater in the SRL group postoperatively. Our study suggests that rATG induction and SRL maintenance immunosuppression facilitate renal recovery for liver transplant recipients that develop acute kidney injury in the early postoperative period.

## 1. Introduction

Renal dysfunction following orthotopic liver transplant (OLT) is a common, posttransplant complication with a 5-year cumulative incidence of 18.1% and is associated with significant morbidity and mortality [1–3]. Although calcineurin inhibitors such as tacrolimus (TAC) and cyclosporine have improved patient and graft survival over the past decade, their use can be associated with significant acute reversible and chronic irreversible nephrotoxicity [4]. In order to prevent calcineurin inhibitor induced nephrotoxicity in the early postoperative period, rabbit antithymocyte globulin induction has also been used in liver transplant recipients to preserve renal function by delaying TAC administration [5].

Sirolimus (SRL), a mTOR inhibitor, is an alternative maintenance immunosuppressant used in liver transplantation [6]. SRL has been associated with a lower incidence of nephrotoxicity when compared to calcineurin inhibitors but may present other adverse effects such as dyslipidemia, myelosuppression, edema, impaired wound healing, and nephrotic range proteinuria [7].

Numerous studies have reported that using SRL following OLT can be both safe and efficacious. In these studies, conversion from calcineurin inhibitors to SRL resulted in stabilization or limited improvement of renal function [8–13]. In addition, the conversion studies describing SRL therapy without concomitant calcineurin inhibitors often delayed initiation of SRL until after the early postoperative period due to concerns of hepatic artery thrombosis [14–16]. Therefore, these patients were essentially converted from calcineurin inhibitors to SRL after postoperative day 30 and did not receive rATG induction.

The primary objective of our study is to determine if SRL MIS and rATG induction are beneficial for liver transplant recipients that develop acute kidney injury in the early postoperative period.

## 2. Materials and Methods

This single-center, retrospective study was conducted at Methodist University Hospital Transplant Institute. Adult OLT recipients were identified from a prospectively maintained liver transplant database from April 6, 2006, to January 3, 2009. This study was conducted in compliance with the Methodist Healthcare Institutional Review Board requirements.

All adult OLT recipients undergoing primary transplant with 1-year follow-up after transplant were included for analysis of renal function. Recipients of any previous transplant and combined liver and kidney transplants were excluded. Patients with less than 1-year follow-up but who met all other inclusion and exclusion criteria were included in the evaluation of patient and graft survival.

The Methodist University Hospital Transplant Institute steroid-free maintenance immunosuppression protocol consists of rATG induction therapy (total dose of 3 mg/kg) and 500 mg of methylprednisolone as a premedication for the first dose of rATG, followed by dual maintenance therapy consisting of either TAC or SRL and mycophenolate mofetil or mycophenolic acid. TAC 1 or 2 mg twice daily was initiated on postoperative days 3–7 if the serum creatinine of the patient was <2.5 mg/dL with initial target drug levels of 6–8 ng/mL. If the serum creatinine of the patient was ≥2.5 mg/dL on postoperative day 7, SRL 2–5 mg daily was initiated to achieve an initial target level of 6–8 ng/mL. Deviations on initiation of TAC or SRL from the protocol were based on physician preference. Patients were categorized according to whether TAC was initiated and continued for a minimum of 30 days as maintenance immunosuppression (MIS) or SRL was initiated or converted from tacrolimus for acute kidney injury within the first 30 days postoperatively. After patients were categorized, if a patient assigned to the TAC or SRL groups received the opposing maintenance immunosuppressant (TAC or SRL) as the primary agent for >14 days, the patient was excluded.

The primary endpoint of this study was to compare the renal function during the first year after transplant between patients receiving TAC or SRL maintenance immunosuppression following OLT. Secondary endpoints included first-year progression to renal replacement therapy (RRT) after transplant after primary hospitalization, incidence of acute cellular rejection (ACR), and patient and graft survivals.

Renal function was evaluated using both the serum creatinine and the eGFR. Serum creatinine was evaluated before transplant; on the date of MIS initiation; and at months 1, 2, 3, 6, and 12. The eGFR was calculated using the abbreviated Modification of Diet in Renal Disease equation [17]. Renal dysfunction was defined as an eGFR less than 60 mL/min/1.73 m for 3 months as defined by Kidney Disease Outcomes Quality Initiative (KDOQI) [18]. Length of stay for primary hospitalization was defined as the time of hospitalization from the day of liver transplantation to the day of discharge. Other data collected included baseline demographics, blood chemistries, immunosuppressant drug levels, and hepatitis C status (+/−).

Differences between the two arms of the study were analyzed by statistical software JMP (SAS Institute, Cary, NC, USA). Student's *t*-test was used for continuous variables, and Fisher's exact test was used for categorical variables. For nonparametric variables, Wilcoxon rank sum test was utilized. All statistical tests were two-tailed with significance defined as a *P* < 0.05. Grubb's test was used to determine outliers in dataset. Further statistical tests were not performed for categories where outliers were identified. Patients with less than 1-year follow-up were included in evaluating patient and graft survivals by using the Kaplan-Meier survival method.

## 3. Results

A total of 197 patients met inclusion criteria with 168 patients (85%) receiving TAC and 29 (15%) receiving SRL. Baseline demographics are listed in Table 1. There was no significant difference in hepatitis C (49% TAC versus 31% SRL) and pretransplant RRT (2% TAC versus SRL 0%) patients between treatment groups. However, significantly more patients in the SRL group displayed pretransplant renal dysfunction (59% SRL versus 21% TAC; *P* < 0.05) and RRT during the primary hospitalization for liver transplantation (14% SRL versus 1% TAC; *P* < 0.05). The median length of stay for the primary hospitalization was increased in the SRL group (*P* < 0.05).

**Figure 1:**
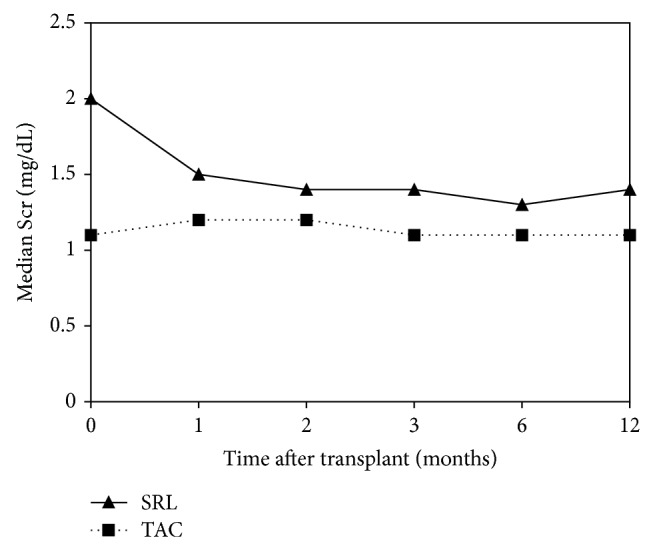
Comparison of mean serum creatinine between SRL (*n* = 29) and TAC (*n* = 168) groups over 1-year follow-up after transplant (*P* < 0.05 at all time points).

**Figure 2:**
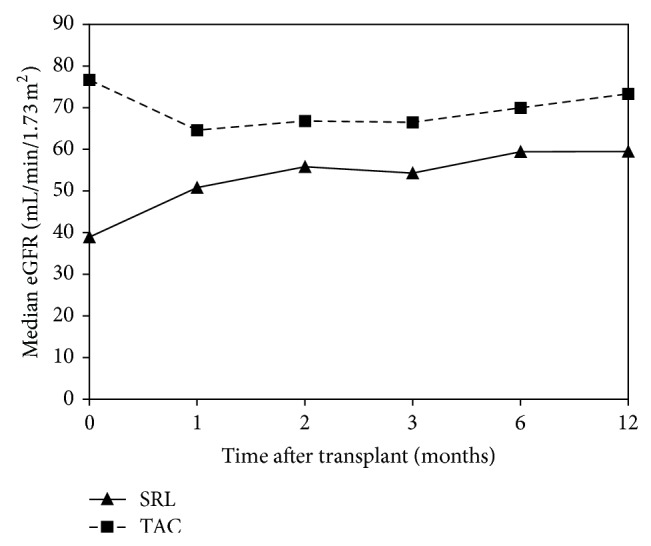
Comparison of estimated glomerular filtration rate between SRL (*n* = 29) and TAC (*n* = 168) groups over 1-year follow-up after transplant (*P* < 0.05 at months 0, 1, 3, 6, and 12).

**Figure 3:**
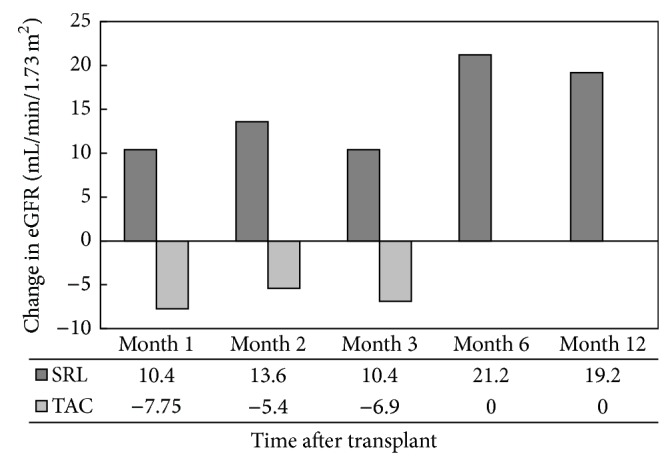
Change in eGFR over 1-year follow-up after transplant from the date of maintenance immunosuppression initiation (*P* < 0.05 at all time points).

Renal function was compared using both serum creatinine and the eGFR. Serum creatinine was significantly elevated, and conversely, eGFR significantly decreased in patients receiving SRL on the day of MIS initiation. Serum creatinine and eGFR values converged in month 2 and then began to stabilize in months 3–12, respectively (Figures 1 and 2). At 1-year follow-up, serum creatinine and eGFR between groups were significantly different (*P* < 0.05). The change in eGFR from the initiation of immunosuppression to 1, 2, 3, 6, and 12 months after transplant showed a significant increase at each time point in the SRL group as compared to the decrease in eGFR seen in the TAC group (Figure 3). The change in eGFR reflects a sustained improvement in renal function over the first year after transplant in the SRL group, and no patients progressed to renal replacement therapy after discharge from primary hospitalization (Table 2).

There was a significantly higher incidence of ACR at 1 year in the SRL group (34% SRL versus 14% TAC); however, there was no significant difference observed in graft survival (82.6% SRL versus 90.7% TAC) (Table 2). Reasons for graft loss included chronic ductopenic rejection and hepatic artery thrombosis. The incidence of hepatic artery thrombosis between the SRL (*n* = 1) and TAC groups (*n* = 5) among patients with graft loss was not significant (*P* > 0.05). However, patient survival was clinically and statistically different between both groups. The TAC group (95.6%) was higher as compared to the SRL group (84.8%). Reasons for patient mortality included sepsis, respiratory failure, small cell lung cancer, and congestive heart failure.

## 4. Discussion

The development of acute kidney injury and chronic kidney disease after transplantation of a nonrenal organ is a significant posttransplant issue. Previous studies have also shown kidney injury to be present in end-stage liver disease patients, despite normal serum creatinine levels [18–20]. Liver disease patients are at an increased risk of kidney injury due to hypoperfusion, deposition of IgA, and hepatitis C virus. Calcineurin inhibitors are the primary immunosuppressants used for liver transplant and have shown benefit for both patient and graft survivals. However, calcineurin inhibitor induced vasoconstriction potentiates the development of acute kidney injury and chronic kidney disease after transplant. Our study demonstrated that a SRL based immunosuppression regimen results in a sustained improvement in renal function with comparable graft outcomes when compared to TAC-based regimens. In addition, 55% of the patients in the SRL group were converted from TAC for acute kidney injury in the early postoperative period. SRL was initiated, on average, by postoperative day 10, which departed from our institutional protocol. This protocol deviation occurred due to the fact that 55% of the patients in the SRL group were converted from TAC for acute kidney injury in the early postoperative period. These patients were converted to SRL, on average, by postoperative day 14 whereas patients initiated on SRL were started by postoperative day 7. However, the current study is distinct from previous studies in which the introduction of SRL was delayed until after postoperative day 30 [8–16]. Recently, a randomized prospective trial evaluating* de novo* SRL was published; however, distinct differences were present such as the following: a loading dose was used for SRL, TAC was given in addition to SRL for maintenance immunosuppression, and rabbit antithymocyte globulin induction was not utilized [21]. As a result, our study is the first study to date evaluating renal effects of SRL therapy with rabbit antithymocyte globulin induction in OLT recipients during the early postoperative period.

In evaluating mean serum creatinine and eGFR over the 12-month follow-up period, we made several interesting observations. Initially, serum creatinine decreased and eGFR improved in the SRL group from the date of immunosuppression initiation to 12 months after transplant. Both the serum creatinine and eGFR improvement were sustained for the remainder of the follow-up period. Conversely, serum creatinine increased and eGFR decreased over that same time period in the TAC group, but eGFR returned toward baseline at the end of follow-up. One possible reason that serum creatinine may not have continued to decrease in the SRL group was due to the addition or conversion to TAC for treatment of ACR. A total of 10 (34%, *n* = 29) SRL patients received TAC for treatment of ACR at some point during follow-up. Addition or conversion to TAC may have prevented any further benefit from SRL and may account for the fact that the only significant difference in mean eGFR between treatment groups was observed at baseline and at month 12.

It is important to consider the baseline characteristics of the treatment groups when evaluating renal function. Patients were started on SRL according to the institutional immunosuppression protocol. As a result, patients in the SRL group were predisposed to having worse renal function as compared to those patients treated with TAC, a fact confirmed by the median eGFR at immunosuppression initiation, which was significantly lower in the SRL group. A higher percentage of the SRL patients also had pretransplant renal dysfunction. Fifty-five percent of patients (*n* = 19) in the SRL group were initiated on TAC and converted to SRL in the early postoperative period. Of these patients, 37% had pretransplant renal dysfunction which reiterates some of the challenges in predicting renal dysfunction after transplant. In our study, the conversion occurred within 30 days after transplant and a loading dose was not given unlike the Sirolimus Liver Conversion Trial. Even though the trial evaluated renal function, the primary endpoints were safety, tolerability, and efficacy of SRL conversion whereas our study focused on renal function after transplant [16]. Even though significantly more SRL patients required RRT during the primary hospitalization, no patients required RRT after discharge from the hospital to the 12-month follow-up period. This may reflect the beneficial effect of SRL in preventing progression to dialysis despite the significant presence of pretransplant renal dysfunction identified from the baseline characteristics. In addition, Ojo et al. showed that acute renal failure postoperatively was associated with increased risk for chronic renal failure. Furthermore, chronic renal failure after transplant was associated with an increased risk of mortality [1]. Collectively, these factors may explain why renal function remained worse in the SRL group despite significant improvement from baseline.

The majority of published studies evaluating the use of SRL in liver transplantation focus on either conversion from or minimization of calcineurin inhibitors. Reasons for conversion include renal dysfunction, intolerable adverse events to calcineurin inhibitors, and diagnosis of hepatocellular carcinoma. Conversion occurred either abruptly or over a long period of time, with conversion anywhere from 3 months to years after transplant [8–16]. Studies involving* de novo* SRL used either calcineurin inhibitors in combination with SRL or delayed SRL therapy beyond the early postoperative period [21–23]. These studies found stabilization of renal function with only limited improvement. These results were also confirmed in a meta-analysis with the majority of trials consisting of a delayed conversion to SRL [24]. However, conversion of tacrolimus to a calcineurin-free regimen (SRL and mycophenolate mofetil) was used in the Spare-the-Nephron Trial [15]. And similar to our study, the trial showed an improvement in renal function during the follow-up period.

In our study, we saw a significant improvement in renal function that was sustained through 1-year follow-up. There are several possible explanations for this difference. In the previously mentioned studies, patients were exposed to calcineurin inhibitors and its nephrotoxicity, both acute and chronic. Therefore, concomitant use of SRL or conversion to SRL may only stabilize renal function and prevent further nephrotoxicity. Due to the early introduction of SRL in our study, patients had minimal or no exposure to TAC. As a result, improvement in renal function in the SRL group was more pronounced.

The incidence of ACR was significantly higher in the SRL group, a finding consistent with other studies of SRL in liver transplantation [15, 16]. However, despite a higher rejection incidence, there was no statistical difference in 1-year graft survival between treatment groups, and the SRL patients responded to acute rejection treatment. It is also important to note that there was no statistical difference in the incidence of hepatic artery thrombosis between treatment groups, which is consistent with recent literature [22, 23, 25, 26].

Despite differences in graft and patient survival rates between groups, a statistical difference was noted in patient survival. Even though this was not a primary endpoint, we hypothesized that a statistical difference would occur, as a higher percentage of patients in the SRL group had pretransplant renal dysfunction, which has been shown to be associated with significant morbidity and mortality after liver transplant [27–30]. This is evident in the median hospital length of stay for the primary hospitalization, which was twice as long in the SRL group as compared to the TAC group.

There were several limitations to this single-center retrospective chart review study. Data collection occurred prospectively but also depended on available information in the electronic and clinic records. As this was not a controlled study, patients could have been interchanged between TAC and SRL based on clinical practice, and immunosuppression selection was dictated by institutional protocol. However, after further review, it was noted that 100% of patients in the SRL groups continued SRL maintenance immunosuppression during the follow- up period for a mean duration of 354 days. It is also important to note that increased hepatic function from the liver transplant will also contribute to improvements in renal function reported in our study. But, as mentioned previously, 55% of the patients in the SRL group experienced acute kidney injury after liver transplant requiring the need for conversion to SRL per our institutional protocol, and the peak increase in eGFR from baseline was noted in the 6th month after transplantation. Our study showed that early SRL conversion provided additional benefit to increasing eGFR after liver transplant. Long-term follow-up in a randomized controlled study utilizing SRL in the early postoperative period would be more meaningful in order to determine whether the benefit of SRL on renal function is sustained after 1-year follow-up. However, this is not likely due to the continued concern of hepatic thrombosis with SRL in liver transplant recipients in the early postoperative period [21]. Routine monitoring of proteinuria, not part of our institutional protocol, is another limitation for our study. Even though this may have a role in renal recovery after transplant, early initiation of SRL facilitated the improvement of renal function.

## 5. Conclusions

Our study suggests that rATG induction and utilization of SRL maintenance immunosuppression in OLT recipients with renal dysfunction may be beneficial in improving renal function as soon as 1 month after transplant. Our study provides another step toward addressing the question concerning the utilization of sirolimus to provide renal benefit for liver transplant recipients with acute kidney injury in the early postoperative period.

## Figures and Tables

**Table 1:** Baseline demographics and secondary endpoints.

	Tacrolimus (*n* = 168)	Sirolimus (*n* = 29)
Recipient characteristics (mean ± SD unless otherwise specified)		
Age at transplant (years)	53 ± 9	54 ± 9
Gender (male), *n* (%)	118 (70)	19 (65)
Race (Caucasian), *n* (%)	128 (76)	18 (62)
BMI (kg/m^2^)	29 ± 5	29 ± 5
Primary disease of hepatitis C, *n* (%)	83 (49)	9 (31)
Model for end-stage liver disease (MELD)	21 ± 6	23 ± 6
Pretransplant renal dysfunction	35 (21)^b^	17 (59)^b^
RRT during primary hospitalization, *n* (%)	1 (1)	4 (14)
Primary hospitalization/length of stay (median days)	7^a,b^	15^a,b^
Mean duration of maintenance immunosuppression therapy (days)	361 ± 2	354 ± 6

^a^Outlier detected using Grubb's test.

^b^ *P* < 0.05 between treatment groups.

**Table 2:** Overall secondary endpoints.

	Tacrolimus (*n* = 168)	Sirolimus (*n* = 29)
Progression to RRT, *n* (%)	0	0
Biopsy-proven acute cellular rejection, *n* (%)	24 (14)^b^	10 (34)^b^
Graft survival 1 year after transplant, %	90.7^c^	82.6^c^
Patient survival 1 year after transplant, %	95.6^b,c^	84.8^b,c^

^a^Outlier detected using Grubb's test.

^b^ *P* < 0.05 between treatment groups.

^c^Survival analysis: *n* = 182 for tacrolimus group and *n* = 46 for sirolimus group.

## Abbreviations

ACR: Acute cellular rejection
eGFR: Estimated glomerular filtration rate
HCV: Hepatitis C virus
MIS: Maintenance immunosuppression
OLT: Orthotopic liver transplant
rATG: Rabbit antithymocyte globulin
RRT: Renal replacement therapy
SRL: Sirolimus
SD: Standard deviation
TAC: Tacrolimus.
